# Antibody Constant Region Peptides Can Display Immunomodulatory Activity through Activation of the Dectin-1 Signalling Pathway

**DOI:** 10.1371/journal.pone.0043972

**Published:** 2012-08-27

**Authors:** Elena Gabrielli, Eva Pericolini, Elio Cenci, Claudia Monari, Walter Magliani, Tecla Ciociola, Stefania Conti, Rita Gatti, Francesco Bistoni, Luciano Polonelli, Anna Vecchiarelli

**Affiliations:** 1 Microbiology Section, Department of Experimental Medicine and Biochemical Sciences, University of Perugia, Perugia, Italy; 2 Microbiology Section, Department of Pathology and Laboratory Medicine, University of Parma, Parma, Italy; 3 Histology Section, Department of Experimental Medicine, University of Parma, Parma, Italy; Federal University of São Paulo, Brazil

## Abstract

We previously reported that a synthetic peptide with sequence identical to a CDR of a mouse monoclonal antibody specific for difucosyl human blood group A exerted an immunomodulatory activity on murine macrophages. It was therapeutic against systemic candidiasis without possessing direct candidacidal properties. Here we demonstrate that a selected peptide, N10K, putatively deriving from the enzymatic cleavage of the constant region (Fc) of human IgG_1_, is able to induce IL-6 secretion and pIkB-α activation. More importantly, it causes an up-regulation of Dectin-1 expression. This leads to an increased activation of β-glucan-induced pSyk, CARD9 and pIkB-α, and an increase in the production of pro-inflammatory cytokines such as IL-6, IL-12, IL-1β and TNF-α. The increased activation of this pathway coincides with an augmented phagocytosis of non opsonized *Candida albicans* cells by monocytes. The findings suggest that some Fc-peptides, potentially deriving from the proteolysis of immunoglobulins, may cause an unexpected immunoregulation in a way reminiscent of innate immunity molecules.

## Introduction

Antibodies (Abs) consist of one or more copies of a tetrameric unit, composed of two heavy (H) and two light (L) chains linked by disulfide bonds [1]. H and L chains comprise variable (V_H_, V_L_) and constant (C_H_, C_L_) regions, folded into functional domains, involved in antigen (Ag) recognition and effector functions respectively. Within the V_H_ and V_L_ domains, 3 hypervariable complementarity determining regions (CDRs) can be identified, and 4 relatively conserved framework regions [2], [3]. In previous studies we demonstrated that the synthetic decapeptide KP, derived from V_L_ of a recombinant antiidiotypic Ab (KT-scFv) representing the internal image of a *Pichia anomala* killer toxin (KT), may exert a microbicidal effect *in vitro* and *in vivo* against a number of pathogenic microorganisms [4], [5]. It displayed inhibitory activity against HIV-1 and influenza A virus, and proved to modulate immune cell function by different mechanisms of action [6], [7], [8].

Synthetic peptides, with sequences identical to CDRs of monoclonal (m)Abs directed to unrelated Ags, showed different antitumor, antiviral and antifungal activities *in vitro* and/or *in vivo*, conceivably involving different mechanisms of action [9].

Moreover, we recently demonstrated that CDR H3 (V_H_CDR3) of a mouse IgM mAb (MoA) specific for difucosyl of human blood group A displayed a therapeutic effect against systemic candidiasis. Given that it did not possess direct candidacidal properties, the observed effect was ascribed completely to its immunomodulatory activity [10].

We then reported that synthetic peptides, representative of sequences included in the constant region (Fc) of the major classes of human immunoglobulins (IgGs), and potentially deriving in significant amounts from their enzymatic cleavage, had a therapeutic effect on experimental mucosal and systemic candidiasis in mouse models [11]. The aim of this study was to evaluate whether such synthetic Fc-peptides have immunomodulatory activity and whether they can play a role in fighting fungal infections.

## Materials and Methods

### Cell Culture Media

RPMI-1640 with L-glutamine and fetal calf serum (FCS) were obtained from Gibco BRL (Paisley, Scotland). Penicillin-Streptomycin solution was obtained from Sigma Aldrich (St. Louis, MO). Complement was inactivated by heating FCS at 56°C for 30 min in a water bath. All reagents and media were negative for endotoxin, as assessed by *Limulus* amebocyte lysate assay (QCL-1000, BioWhittaker, Walkersville, MD).

### Fc-peptides and Negative Control Peptide

Fc-peptides derived from the amino acid sequence analysis of the different classes of Abs with a bioinformatic approach [11] were chemically synthesized to be used for *in vitro* studies of immunomodulatory activity. The Fc-peptides are listed in Table 1.

An irrelevant synthetic decapeptide (MSTAVSKCAT), previously proven to be devoid of either fungicidal or immunomodulatory activity *in vitro,* was synthesized to be used as a negative control (NC) [4], [7], [10].

**Table 1:** Characteristics of Fc-peptides.

Fc-peptides	Ig class	Sequences
H4L	IgG, IgM, IgA	HEAL
N10K	IgG_1_	NQVSLTCLVK
S8K	IgG_1_	SLSLSPGK
G7R	IgM	GFPSVLR
T11F	IgM	TCRVDHRGLTF

### Fungal Strain

The origin and characteristics of the highly virulent *Candida albicans* strain (CA-6) used in this study have previously been described [12]. The culture was maintained by serial passages on Sabouraud agar (BioMérieux, Lyon, France). The yeast cells were harvested by suspending a single colony in saline, washed twice, counted in a hemocytometer and adjusted to the desired concentration. *C. albicans* cells were inactivated by heating at 60°C for 30 min (HI CA-6).

### Cell Separation

Heparinised venous blood was obtained from healthy donors and diluted with RPMI-1640 medium. The peripheral blood mononuclear cells (PBMC) and neutrophils were obtained by density gradient centrifugation on Ficoll-Hypaque. To obtain monocytes, PBMC were washed twice in RPMI-1640 medium, and incubated for 1 h at 37°C plus 5% CO_2_ in a culture flask. After 1 h of incubation, adherent cells (monocytes) were recovered as described by Monari C. *et al*. [13]. Ethics approval for this study was obtained from “Comitato Etico Aziende Sanitarie” Umbria (CEAS) N° 1874/11. Written informed consent was obtained from all subjects prior to sample collection.

**Figure 1:**
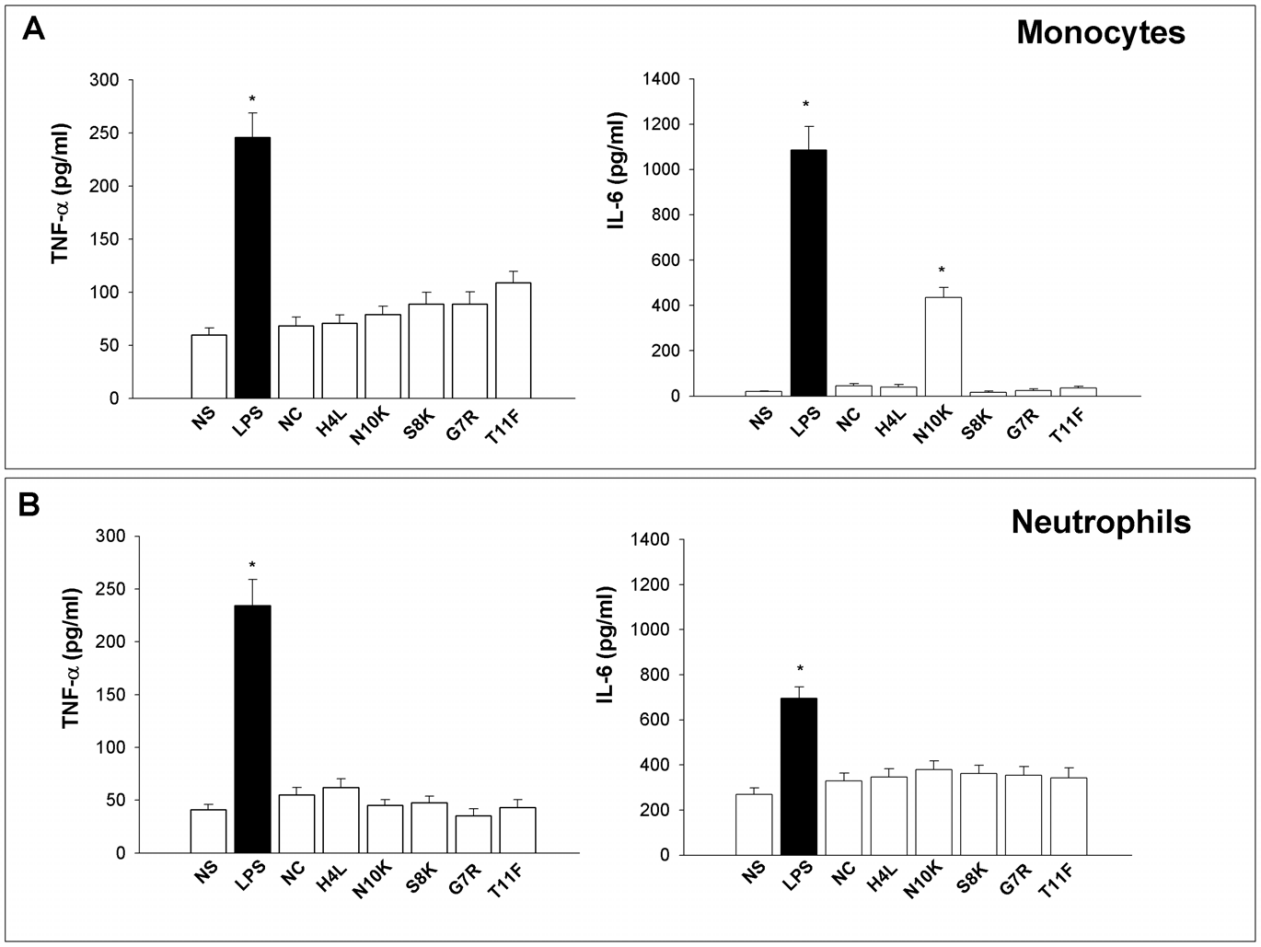
TNF-α and IL-6 production by monocytes and neutrophils stimulated with Fc-peptides. Monocytes (A) or neutrophils (B) (both 1×10^7^/ml) were cultured in the presence/absence (non stimulated, NS) of LPS, NC or different Fc-peptides (all 10 µg/ml) for 18 h. After incubation, TNF-α and IL-6 levels were evaluated in culture supernatants using specific ELISA assays. *, *P*<0.05 (n = 5; treated *vs* untreated cells). Error bars denote s.e.m.

### Cytokine Production

Human neutrophils and monocytes (1×10^7^/ml) were incubated for 18 h in RPMI-1640+10% FCS with heat inactivated complement (complete medium) at 37°C plus 5% CO_2_ in the presence/absence of LPS, NC or different Fc-peptides (H4L, N10K, S8K, G7R and T11F, all 10 µg/ml). After incubation, the supernatants were collected and tested for TNF-α and IL-6 levels by specific ELISA assays (Biosource, Camarillo, CA). Monocytes (5×10^6^/ml) were pre-incubated for 30 min in complete medium at 37°C plus 5% CO_2_ in the presence/absence of NC, N10K (both 10 µg/ml) or piceatannol (100 µM), and then stimulated for 18 h in the presence/absence of HI CA-6 (E/T: 1/10), curdlan (25 µg/ml) or zymosan (50 µg/ml). After incubation, the supernatants were collected and tested for IL-6, IL-12p40, TNF-α, and IL-1β levels by specific ELISA assays (Biosource). In selected experiments, monocytes (5×10^6^/ml) were stimulated for 6 h, 18 h or 48 h in the presence/absence of zymosan (100 µg/ml), NC or N10K (both 10 µg/ml). After incubation, the supernatants were collected and tested for TNF-α and IL-1β levels by specific ELISA assays (Biosource).

**Figure 2:**
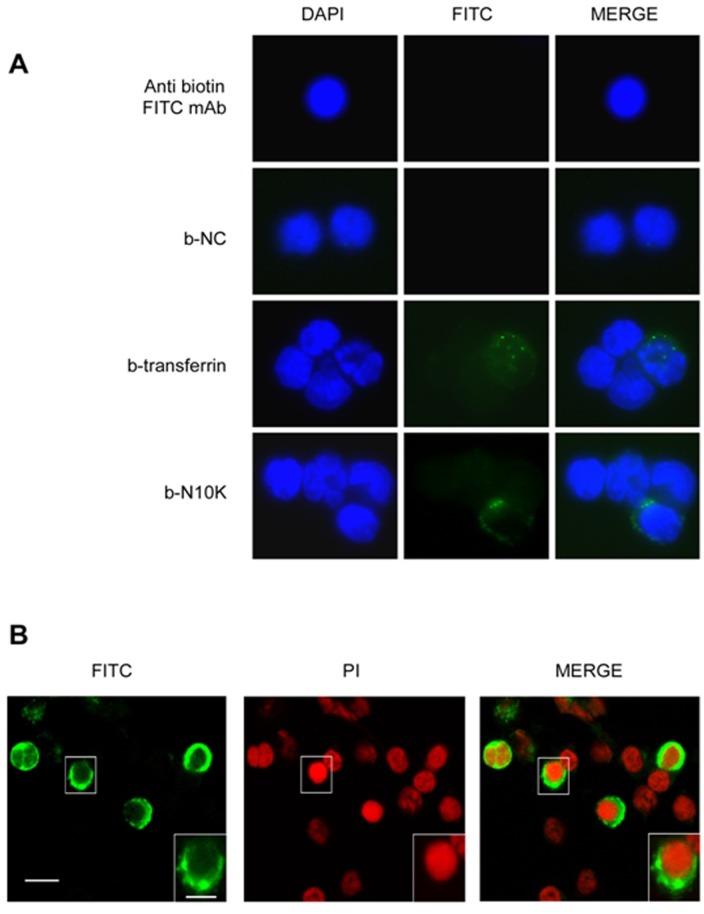
Endocytosis of N10K. Monocytes (1×10^6^/ml) were incubated for 30 min with b-transferrin (25 µg/ml), b-NC or b-N10K (both 10 µg/ml). After incubation, permeabilized cells were reacted with FITC-labelled mAb to biotin, stained with DAPI and subsequently examined under fluorescent light microscopy (A). In selected experiments, monocytes (1×10^6^/ml) were cultured with b-N10K (10 µg/ml). After incubation, permeabilized cells were stained with S-FITC and PI and subsequently examined by confocal microscopy (B). The images show a single equatorial section of cells. In the insets, one cell at large magnification is shown. Bar = 10 µm,. Inset bar = 5 µm.

**Figure 3:**
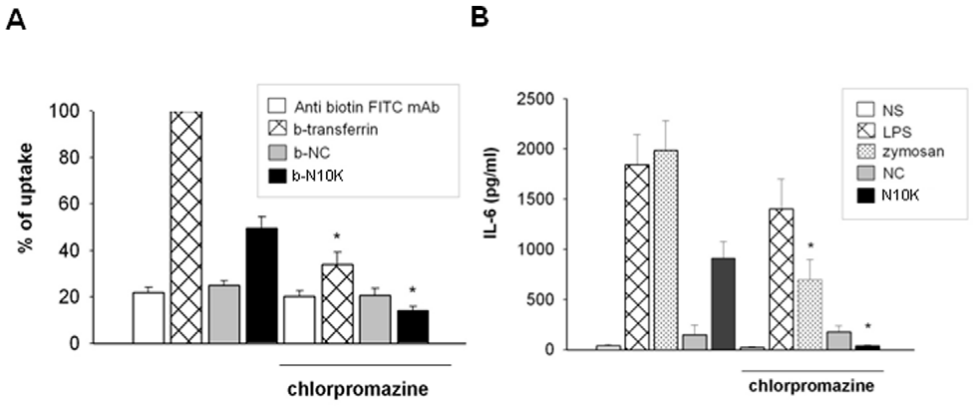
Inhibition of N10K endocytosis. Monocytes (1×10^6^/ml) were pre-treated in the presence/absence of chlorpromazine (10 µg/ml) for 30 min and subsequently treated in the presence/absence (NS) of b-transferrin (25 µg/ml), b-NC or b-N10K (both 10 µg/ml) for 30 min. After incubation, permeabilized cells were stained with FITC-labelled mAb to biotin and analyzed by FACScan flow cytometry. Data are reported as percentage of uptake (A). *, *P*<0.05 (n = 5; chlorpromazine treated *vs* untreated cells). Error bars denote s.e.m. Monocytes (2×10^7^/ml) were pre-treated in the presence/absence of chlorpromazine (10 µg/ml) for 30 min and subsequently treated in the presence/absence (NS) of zymosan (100 µg/ml), LPS, NC or N10K (all 10 µg/ml) for 18 h. After incubation, IL-6 levels were evaluated in culture supernatants using specific ELISA assays. (B) *, *P*<0.05 (n = 5; chlorpromazine treated *vs* untreated cells). Error bars denote s.e.m.

Cytokine titers were calculated by reference to standard curves, constructed with known amounts of recombinant cytokines.

### Flow Cytometry Analysis

Monocytes (1×10^6^/ml) were incubated for 30 min in complete medium at 37°C plus 5% CO_2_ in the presence/absence of zymosan (100 µg/ml), NC, N10K, H4L, S8K, G7R or T11F (all 10 µg/ml). In selected experiments, monocytes (1×10^6^/ml) were pre-incubated for 30 min in complete medium at 37°C plus 5% CO_2_ in the presence/absence of NC, N10K, H4L, S8K, G7R or T11F (all 10 µg/ml) and subsequently incubated for 3 h in the presence/absence of zymosan (50 µg/ml). After incubation, cells were fixed with 4% formalin for 5 min at room temperature (RT), washed twice with fluorescence buffer (FB), and labelled for 45 min on ice with R-Phycoerythrin (RPE) conjugated mAbs to human Dectin-1 (5 µl/test; Mouse IgG_2b_ isotype) (AbD Serotec, Oxford, UK), TLR-2 (10 µl/10^6^ cells; Mouse IgG_1_ isotype) (USBiological, Massachusetts, MA) and TLR-4 (5 µl/test; Mouse IgG_2a,kappa_ isotype) (eBioscience, San Diego, CA). Then cells were washed twice with FB and analyzed using a FACScan flow cytometer (BD Biosciences, Franklin Lakes, NJ). To evaluate phosphorylation of IkB-α, cells (1×10^6^/ml) were incubated for 30 min in complete medium at 37°C plus 5% CO_2_ in the presence/absence of zymosan (100 µg/ml), NC or N10K (both 10 µg/ml). After incubation, cells were fixed with 1.5% formalin for 5 min at RT, washed, incubated with absolute methanol (500 µl/10^6^ cells) for 10 min on ice to permeabilize cells, washed twice and incubated with goat polyclonal Ab to phospho-IkB-α (dilution 1∶50, Santa Cruz Biotechnology Inc., Santa Cruz, CA) followed by Cy3-labelled secondary Ab (dilution 1∶100, Chemicon Int., Temecula, CA) [14]. Data are expressed as mean of fluorescence intensity (MFI) of labelled cells. Autofluorescence was assessed using untreated cells. Control staining of cells with an irrelevant Ab was used to obtain background fluorescence values.

**Figure 4:**
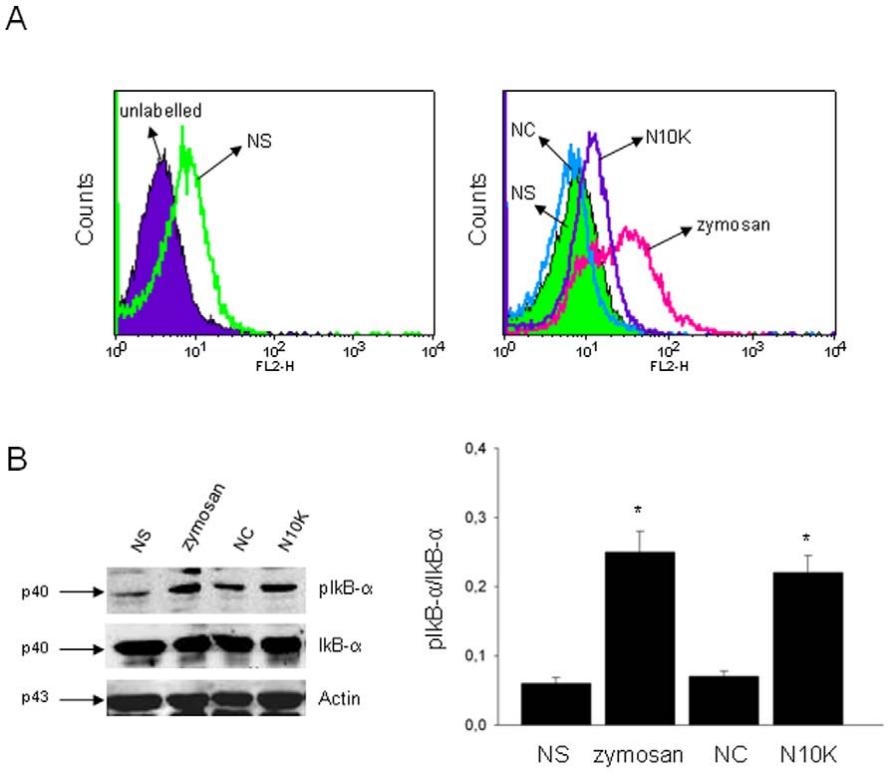
Activation of IkB-α. Monocytes (1×10^6^/ml) were cultured in the presence/absence (NS) of zymosan (100 µg/ml), NC or N10K (both 10 µg/ml) for 30 min. After incubation, intracellular staining of cells was performed using phospho-IkB-α Ab followed by Cy3-labelled secondary Ab and analyzed by FACScan flow cytometry. FACScan histograms represent MFI (A). Monocytes (3×10^6^/ml) were cultured as above described. After incubation cell lysates were subjected to western blotting. Membranes were incubated with Abs to phospho-IkB-α and IkB-α. Western blotting bands are shown; phospho-IkB-α was normalized against IkB-α (B). *, *P*<0.05 (n = 5; treated *vs* untreated cells). Error bars denote s.e.m.

### Endocytosis of N10K

Monocytes (1×10^6^/ml) were pre-treated for 30 min in complete medium at 37°C plus 5% CO_2_ in the presence/absence of chlorpromazine (10 µg/ml) and subsequently treated for 30 min in the presence/absence of biotin-labelled transferrin (b-transferrin) (25 µg/ml), biotin-labelled NC (b-NC) or biotin-labelled N10K (b-N10K) (both 10 µg/ml) [15]. After incubation, cells were fixed with 4% formalin for 5 min at RT, permeabilized with 0.1% saponin, incubated for 20 min on ice with fluorescein isothiocyanate (FITC)-conjugated mAb to biotin (1 µl/test; Mouse IgG_1_ isotype) (Miltenyi Biotec, Bergish Gladbach, Germany), and analyzed by a FACScan flow cytometer. Data are reported as percentage of uptake.

**Figure 5:**
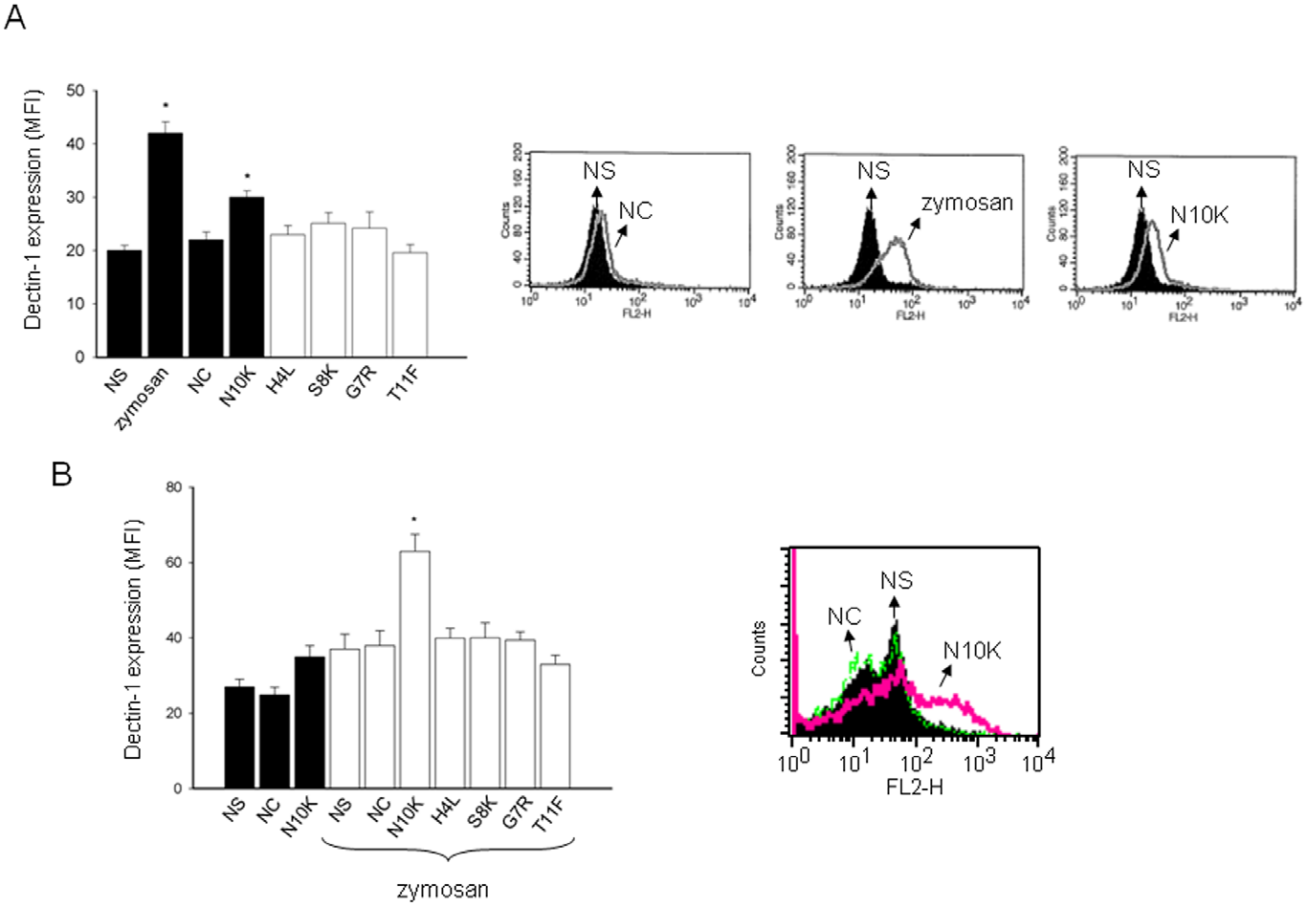
Modulation of Dectin-1 expression by N10K. Monocytes **(**1×10^6^/ml) were incubated for 30 min in the presence/absence (NS) of zymosan (100 µg/ml), NC, N10K, H4L, S8K, G7R or T11F (all 10 µg/ml) (A). In selected experiments, monocytes **(**1×10^6^/ml) were pre-treated for 30 min in the presence/absence (NS) of NC, N10K, H4L, S8K, G7R or T11F (all 10 µg/ml) and subsequently stimulated for 3 h with zymosan (50 µg/ml) (B). After incubation, cells were labelled with RPE mAb to Dectin-1 and analyzed by FACScan flow cytometry. MFI of labelled cells is shown as a histogram or as a FACScan histogram (A and B). *, *P*<0.05 (n = 7; treated *vs* untreated cells). Error bars denote s.e.m.

Monocytes (2×10^7^/ml) were pre-treated for 30 min in complete medium in the presence/absence of chlorpromazine (10 µg/ml) and subsequently treated for 18 h in the presence/absence of zymosan (100 µg/ml), LPS, NC or N10K (all 10 µg/ml). After incubation, the supernatants were collected and tested for IL-6 level using specific ELISA assays. The cytokine titer was calculated by reference to a standard curve, constructed with known amounts of recombinant cytokine.

**Figure 6:**
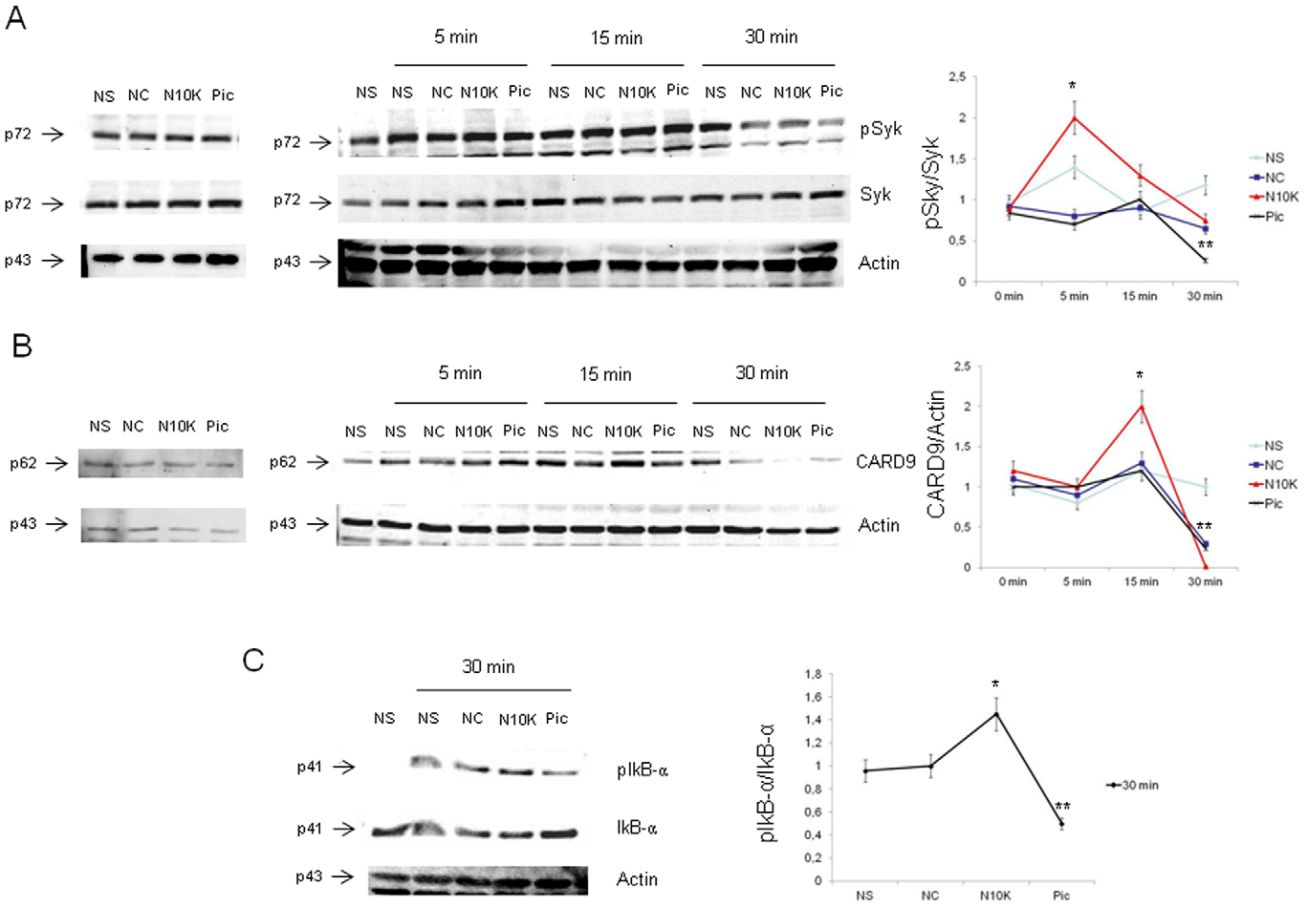
Dectin-1 signalling pathway activation. PBMC (3×10^6^/ml) were pre-treated for 30 min in the presence/absence (NS) of NC, N10K (both 10 µg/ml) or piceatannol (Pic) (100 µM) and subsequently incubated for different times in the presence/absence of zymosan (50 µg/ml). After incubation, cell lysates were subjected to western blotting. Membranes were incubated with Abs to pSyk, Syk, CARD9, Actin, pIkB-α and IkB-α. Western blotting bands and protein normalizations are shown (A, B and C). *, *P*<0.05 (n = 5; N10K treated *vs* untreated cells). **, *P*<0.05 (n = 5; Pic treated *vs* untreated cells). Error bars denote s.e.m.

### Fluorescence Microscopy

Monocytes (1×10^6^/ml) were treated for 30 min in complete medium at 37°C plus 5% CO_2_ in the presence/absence of b-transferrin (25 µg/ml), b-NC or b-N10K (both 10 µg/ml). After incubation, cells were fixed with 4% formalin for 5 min at RT, permeabilized with 0.1% saponin, washed with 0.1% saponin, incubated for 20 min on ice with FITC-conjugated mAb to biotin (Miltenyi Biotec), washed with 0.1% saponin and resuspended with FB. After staining, cells were collected by cytospin (2×10^5^/200 µl) at 700 g for 7 min, reacted with 4′6-diamidino-2-phenylindole (DAPI, Sigma) and subsequently examined under a fluorescent light microscope (Carl Zeiss). An irrelevant Ab was used to obtain background of fluorescence. Each condition was studied in triplicate, and three images were taken for each sample. The figures shown are representative of 5 separate experiments.

**Figure 7:**
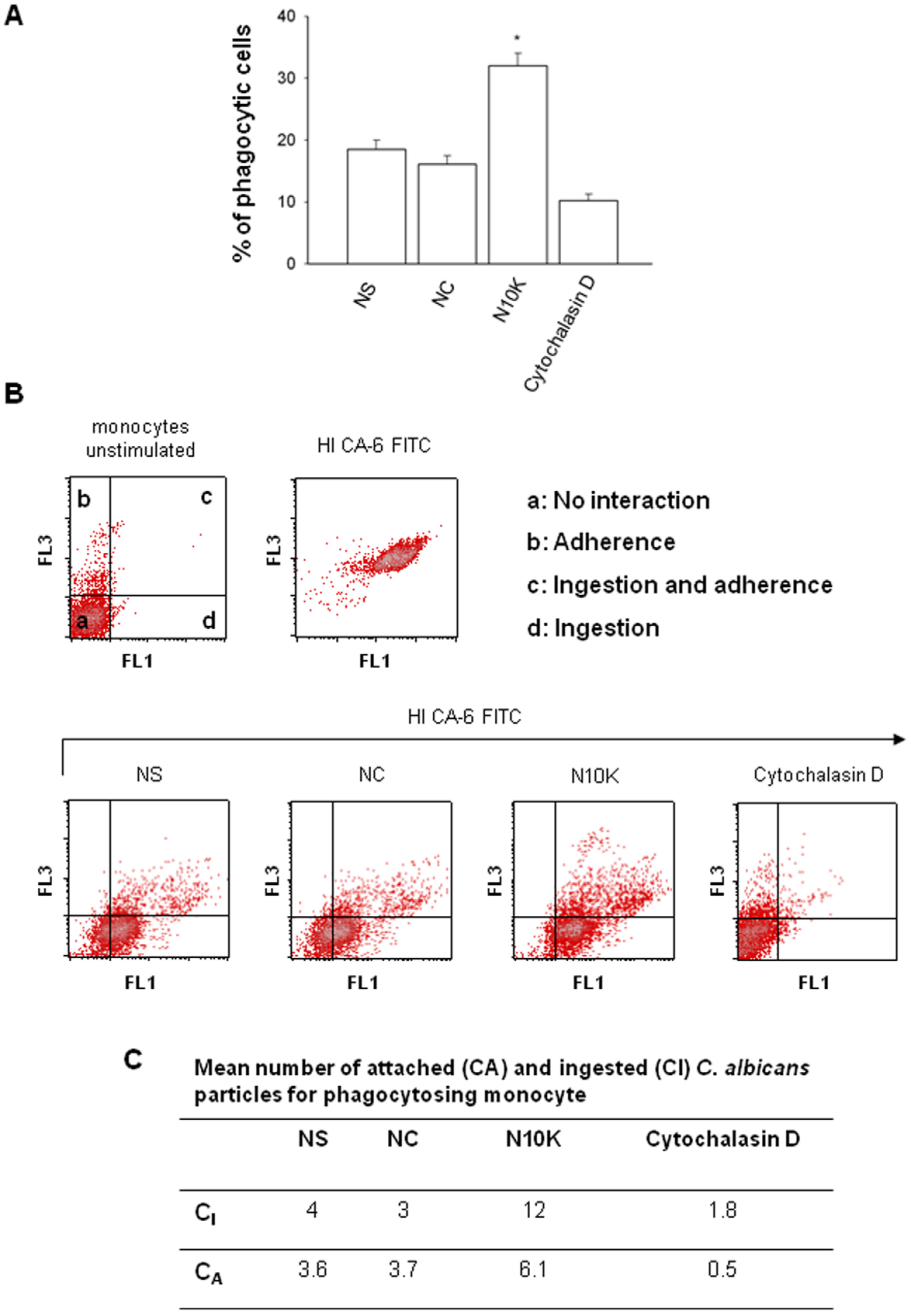
Phagocytosis of *C. albicans* cells. Monocytes (1×10^7^/ml) were incubated for 30 min in the presence/absence of NC, N10K (both 10 µg/ml) or Cytochalasin D (30 µM) and subsequently incubated with heat inactivated FITC-labelled yeast cells (HI CA-6) for 30 min. Percentage of phagocytic cells was determined by flow cytometry (A). *, *P*<0.05 (n = 7; N10K treated *vs* untreated cells). Error bars denote s.e.m. Monocytes (1×10^7^/ml) were treated as above described. Quantification of recognition and ingestion of yeast cells were determined by flow cytometry. Dot plots of the distribution of green (FL1) and red (FL3) fluorescence are shown in panel B. Four distinct cell subsets are identified, which coincide with four types of monocyte-yeast interaction: quadrant a, no interaction; quadrant b, adherence; quadrant c, ingestion and adherence; quadrant d, ingestion (B). Panel C shows the mean number of attached (CA) and ingested (CI) *C. albicans* particles per each phagocytosing monocyte calculated using data from plots of panel B (see Materials and Methods section).

### Confocal Microscopy Studies

Monocytes (1×10^6^/ml) were incubated for 30 min at 37°C plus 5% CO_2_ with b-N10K, washed three times with PBS and fixed with 4% formalin for 10 min at RT. The cells were permeabilized in 0.1% Triton X-100 for 10 min at RT and blocked with PBS plus 2% BSA for 1 h at RT. Permeabilized cells were then incubated for 1 h in the dark with streptavidin-fluorescein isothiocyanate (S-FITC) (Invitrogen, Camarillo, CA) diluted 1∶200 in PBS followed by propidium iodide (PI) (250 µg/ml) (Sigma-Aldrich) staining for 5 min. Cells were washed three times with PBS and 20 µl of mounting medium was added to each spot. Monocytes were examined under a Zeiss LSM 510 Meta confocal microscope with a 100×, 1.30 NA oil objective.

**Figure 8:**
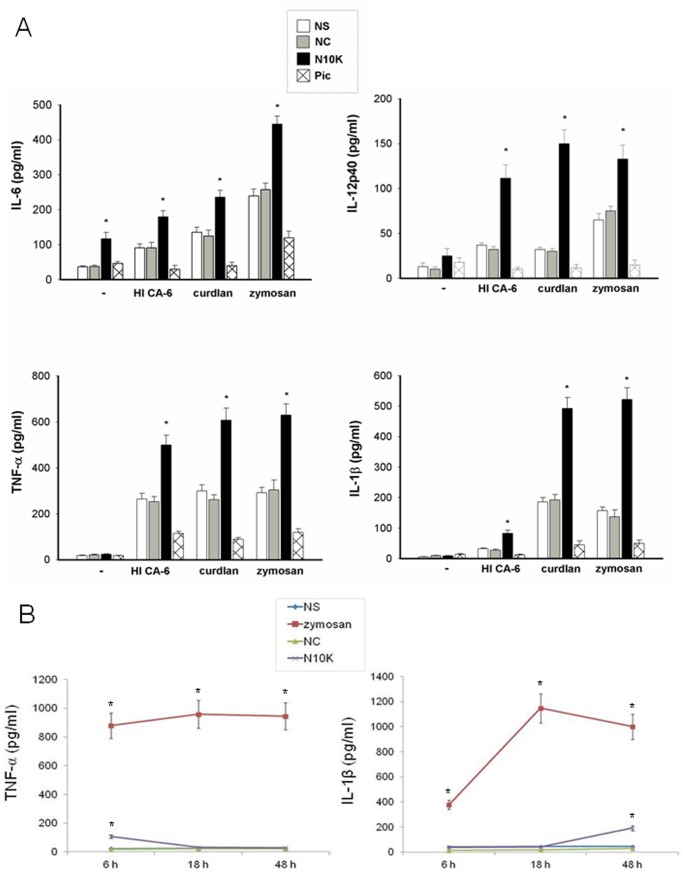
Proinflammatory cytokine production. Monocytes (5×10^6^/ml) were pre-treated in the presence/absence (NS) of NC, N10K (both 10 µg/ml) or piceatannol (Pic) (100 µM) for 30 min and subsequently incubated with heat inactivated *C. albicans* cells (HI CA-6, E/T:1/10), curdlan (25 µg/ml) or zymosan (50 µg/ml) for 18 h. After incubation, cytokine levels were evaluated in culture supernatants using specific ELISA assays. (A) *, *P*<0.05 (n = 5; N10K treated *vs* untreated cells). Error bars denote s.e.m. In selected experiments monocytes (5×10^6^/ml) were treated in the presence/absence (NS) of NC, N10K (both 10 µg/ml) or zymosan (100 µg/ml) for 6 h, 18 h or 48 h. After incubation, cytokine levels were evaluated in culture supernatants using specific ELISA assays. (B) *, *P*<0.05 (n = 5; treated *vs* untreated cells).

S-FITC and PI were excited with 488 nm argon and 543 nm He-Ne laser lines, respectively. Acquisition was carried out in a multitrack mode with the relevant beamsplitters; barrier filters were 505–530 band pass and 560 long pass for the above signals, respectively. A series of x-y sections was acquired with a z-step of 0.5 µm to cover the whole height of the samples.

### Western Blot Analysis

PBMC (3×10^6^/ml) were pre-incubated for 30 min in complete medium at 37°C plus 5% CO_2_ in the presence/absence of NC, N10K (both 10 µg/ml) or piceatannol (100 µM) and subsequently incubated for 5 min, 15 min or 30 min in the presence/absence of zymosan (50 µg/ml). In selected experiments, monocytes (3×10^6^/ml) were incubated for 30 min in complete medium at 37°C plus 5% CO_2_ in the presence/absence of zymosan (100 µg/ml), NC or N10K (both 10 µg/ml). After incubation, cells were washed and lysed with mammalian protein extraction reagent (M-PER) in the presence of protease and phosphatase inhibitors (all from Pierce, Rockford, IL). The protein concentration was determined with a BCA protein assay reagent kit (Pierce). The lysates (40 µg of each sample) were separated by sodium dodecyl-sulfate-10% PAGE, transferred to a nitrocellulose membrane (Pierce) for 1 h at 100 V in a blotting system (Bio-Rad, Hercules, CA) for Western Blot analysis, and the membranes were incubated over-night with rabbit polyclonal Abs to phospho-Syk (pSyk; dilution 1/300) (Abcam), CARD9 (dilution 1/200) (Santa Cruz) and phospho-IkB-α (pIkB-α; dilution 1/1000) (Cell Signalling Technology, Beverly, MA) in blocking buffer. Detection was achieved using the appropriated secondary Abs coupled to HRP, followed by use of a Chemiluminescent kit (Bio-Rad). Immunoblotting with rabbit polyclonal Abs to Syk, Actin and IkB-α (all with a dilution 1/200; all from Santa Cruz) were used as internal loading controls to ensure equivalent amounts of protein in each lane. Immunoreactive bands were visualized and quantified by Chemidoc Instrument (Bio-Rad). A quantitative analysis of the region of interest was performed as previously described [10].

### FITC-labelling of *C. albicans* Cells

For use in the phagocytosis assay, HI CA-6 cells were harvested and the concentration was adjusted to 1×10^8^/ml. Yeast cells were labelled with FITC at 0.5 mg/ml in PBS at RT for 10 min as previously described [16].

### Determination of *C. albicans* Interaction with Monocytes

Monocytes (1×10^7^/ml) were incubated for 30 min in complete medium at 37°C plus 5% CO_2_ in the presence/absence of NC, N10K (both 10 µg/ml) or Cytochalasin D (30 µM) (Sigma) and subsequently 100 µl of each cell suspension were incubated with FITC-labelled yeast cells (100 µl; 1×10^8^/ml) for 30 min at 37°C plus 5% CO_2_. Phagocytosis was stopped by adding 1 ml of ice cold PBS to the suspension. Trypan blue (200 µg/ml) (Sigma) was added and samples were incubated for 10 min to quench fluorescence of non-internalized fungi. Unbound Trypan blue was then removed by centrifugation and the percentage of phagocytic cells was determined by flow cytometry [16], [17]. Monocytes (1×10^7^/ml) were stimulated as above described. After incubation, quantification of recognition and ingestion of *C. albicans* cells was done using flow cytometry analysis as previously described [18].

Trypan blue produces red fluorescence in cells upon binding. This property of Trypan blue together with its ability to quench the green fluorescence of fluorescein-labelled particles makes it possible to simultaneously assess membrane-bound (CA) and ingested (CI) *C. albicans* particles during phagocytosis by human monocytes. Hence, the particles ingested by monocytes will fluoresce green (FL1), while those attached to the cell membrane will fluoresce bright red (FL3). A FSC threshold was set to gate out debris. Monocytes and free *C. albicans* were discriminated by combined measurements of FSC and SSC and gated in R1 and R2 regions, respectively. The percentage distribution of monocyte subsets was calculated from a dot plot analysis (FL1 *vs* FL3) of R1 gated events. The mean number of attached yeast particles per monocytes (CA) was calculated by means of the formula below: C*_A_* = F*_monocyte_*/F*_C_,,* where F*_monocyte_* is the mean red fluorescence in monocytes (calculated considering the monocytes bearing red fluorescent particles, which are the events in quadrant b and c) and F*_C_* is the mean red fluorescence of free *C. albicans* particles. The mean number of ingested yeasts per monocytes (CI) was assessed as follows: C*_I_* = C–C*_A_,* where C is the mean number of monocyte-associated yeasts (i.e. both attached and internalized particles), calculated by subtracting the number of free *C. albicans* at the selected incubation time from the number of free particles at t_0_ divided by the number of fluorescent phagocytosing monocytes (calculated as the sum of the events in quadrants b, c and d). CA and CI show the phagocytic index for each phagocytosing monocyte.

### Statistical Analysis

Data are reported as the mean ± s.e.m. from triplicate samples of 5–7 experiments and were evaluated using ANOVA. Post hoc comparisons were done with Bonferroni’s test. A value of *P*<0.05 was considered significant.

## Results

We have recently demonstrated the immunomodulatory effects of a synthetic peptide, V_H_CDR3, whose sequence was derived from the variable region of a mouse IgM mAb specific for difucosyl of human blood group A [10].

This study evaluated the possibility that fragments selected within the constant region of Abs, which could be obtained upon digestion by physiological enzymes, i.e. trypsin and chymotrypsin, display immunomodulatory activity.

First, the capacity of Fc-peptides to stimulate cytokine production by innate immune cells such as neutrophils and monocytes was evaluated. An irrelevant synthetic peptide was used as a negative control (NC) in our experimental system [7], [10]. LPS was used as a positive control. Our results show that N10K induced a significant up-regulation of IL-6 production, but not of TNF-α production, by monocytes (Fig. 1A), while it was not able to induce an increased production of either cytokine by neutrophils (Fig. 1B). The other Fc-peptides and the NC peptide did not affect cytokine production by either cell type.

Since N10K exerted a stronger effect on IL-6 production by monocytes than all the other peptides, it was chosen for subsequent experiments. It has been demonstrated that endocytosis of light chains of Abs, excessively produced in multiple myeloma by human proximal tubule cells, leads to production of cytokines, such as IL-6, through activation of NF-kB [19]. In the light of this evidence, we hypothesized that IL-6 production could be mediated by the endocytosis process. Our results indicate that N10K was efficiently internalized by monocytes after 30 min as shown by conventional fluorescence (Fig. 2A) or confocal microscopy (Fig. 2B) and cytofluorimetric (Fig. 3) analyses. In particular, N10K shows a cytoplasmic distribution (Fig. 2B and inset). N10K uptake was significantly inhibited by clathrin-dependent endocytosis inhibitor chlorpromazine. Transferrin was tested as an appropriate control of chlorpromazine activity (Fig. 2A and 3) [15], [20]. Indeed chlorpromazine pre-treatment caused significant inhibition of IL-6 production induced by zymosan and N10K treatment. Additionally, chlorpromazine pre-treatment did not affect IL-6 production induced by LPS. NC stimulation did not produce any effects (Fig. 2 and 3).

The activation of IkB-α phosphorylation after N10K treatment was also evaluated in our experimental system. Results in Figure 4 show that 30 min after N10K treatment, there was a significant up-regulation of IkB-α phosphorylation as indicated by flow cytometry (A) and western blot (B) analyses. A significant induction of p-IkB-α activation was observed after stimulation with zymosan. Conversely, NC treatment did not show any effect.

Given that N10K can interact with innate immune cells and induce IL-6 production, we next explored its ability to modulate PRRs expression on monocytes. To this end, TLR-2, TLR-4 and Dectin-1 expression were evaluated after peptide stimulation.

Our results show that there was a significant up-regulation of Dectin-1 expression in monocytes after 30 min of stimulation with N10K but not with NC or other peptides (Fig. 5A). Unlike Dectin-1, TLR-2 and TLR-4 showed no modulation after N10K addition (not shown).

It has been reported that zymosan, predominantly composed of β-glucans, can induce Dectin-1 expression on monocytes [21], [22], [23]. To explore the possibility that N10K increases zymosan-induced Dectin-1 expression, cells were pre-treated for 30 min with peptides and then stimulated for 3 h with zymosan. The results, reported in Figure 5B, show a marked up-regulation of Dectin-1 expression following zymosan addition combined with N10K. No effect was observed after pre-treatment with NC or other peptides.

It is well known that the signal transduction pathway of Dectin-1 occurs via Syk and CARD9 activation [17], [24], [25]. Thus, the possibility that N10K treatment influences the activation of proteins involved in zymosan-induced Dectin-1 signal pathway was considered. To this purpose, PBMC were pre-treated with N10K, NC, or piceatannol, which has been reported to selectively block the Syk transduction pathway [17], [26], [27], [28], and subsequently stimulated with zymosan for 5 min, 15 min or 30 min. The results show that there was a high level of Syk phosphorylation after 5 min of zymosan stimulation in cells pre-treated with N10K, the effect decreasing to negligible levels after 15 min and 30 min of zymosan stimulation (Fig. 6A). This effect was correlated with an increase of the CARD9 protein level and increased phosphorylation of IkB-α in cells pre-treated with N10K and then stimulated with zymosan for 15 min and 30 min respectively (Fig. 6B and C). The specificity of the stimulatory activity of N10K was confirmed by the inability of the NC to activate cells. Piceatannol inhibited pSyk, CARD9 and pIkBα activation after 30 min of zymosan stimulation (Fig. 6).

Dectin-1 is a major receptor for β-glucans expressed on the cell wall of fungi such as *C. albicans* [21], [29]. Dectin-1 is the primary receptor for phagocytosis of non opsonic zymosan particles and fungi such as *C. albicans* by phagocytic cells [30], [31], [32], [33], [34]. As a consequence, the possibility that N10K can affect the recognition of *C. albicans* was evaluated. Phagocytosis of non opsonised HI CA-6 by monocytes pre-treated for 30 min with N10K was evaluated after 30 min of incubation. The results reported in Figure 7A show that the pre-treatment of cells with N10K induced a significant increase in the percentage of phagocytic cells. Moreover, N10K was also able to increase the percentage of yeast particles adhered or ingested by monocytes (Fig. 7B), and this effect was correlated to the increase of the mean number of attached and ingested *C. albicans* particles for each phagocytosing monocyte (phagocytic index) (Fig. 7C). The specificity of N10K activity was confirmed by the inability of the NC to increase *C. albicans* uptake by monocytes. Cytochalasin D was used to block the phagocytic process [18].

Given that the engagement of Dectin-1 can regulate the production of a variety of cytokines, we explored the possible influence of N10K in the production of proinflammatory cytokines in our experimental system. To this end, monocytes were pre-treated with N10K for 30 min and then stimulated for 18 h in the presence/absence of HI CA-6, zymosan, or curdlan, a Dectin-1 specific agonist and a linear polymer made up of β-1,3-glucan [35]. The results show that N10K was able to induce a significant production of IL-6, and when N10K was used in combination with HI CA-6, curdlan and zymosan, a further increase in IL-6, IL-12p40, TNF-α and IL-1β production was observed (Fig. 8A). NC did not affect cytokine production. The treatment with piceatannol caused a significant decrease of cytokine production independently of the stimulus (Fig. 8A). Moreover, the kinetics of TNF-α and IL-1β production by monocytes stimulated with N10K show that N10K is also able to induce TNF-α and IL-1β production after 6 h and 48 h, respectively (Fig. 8B).

## Discussion

It has been demonstrated that, irrespective of the specificity of the native Ab for a given Ag or the isotype, CDR-related or Fc-peptides from murine or human Abs may display *in vitro*, *ex vivo* and/or *in vivo* antimicrobial, antiviral, antitumor and immunomodulatory activity reminiscent of molecules of innate immunity [4], [5], [9], [10], [11], [36].

The aim of this work was to evaluate whether an Fc-peptide, N10K, reputedly deriving from the enzymatic cleavage of the C region of IgG_1_ Abs, can exert immunomodulatory activity on human monocytes *in vitro*. The results of the research demonstrate that N10K is able to induce: i) up-regulation of Dectin-1 expression, and further increase of Dectin-1 expression in combination with microbial particles such as zymosan; ii) activation of pIkB-α; iii) increased activation of β-glucan-induced pSyk, CARD9 and pIkB-α; iv) production of IL-6, TNF-α and IL-1β, and a further increase in the production of IL-6, IL-12p40, IL-1β and TNF-α when used in combination with heat inactivated *C. albicans*, curdlan and zymosan; vi) increased rate of phagocytosis of non opsonized *C. albicans* cells. These data suggest that selected short sequences representative of the Fc of Abs can influence the immune response.

Recently we demonstrated that the Fc-peptide N10K displayed therapeutic activity in a mouse model of systemic and vaginal candidiasis, putatively owing to its demonstrated *in vitro* fungicidal activity. This beneficial effect was evidenced as increase of survival and as acceleration of fungal clearance from the vagina [11]. Here we demonstrate that these important antimicrobial effects are coupled with robust immunoregulatory activity. Indeed our results demonstrate that N10K is rapidly internalized - and visible in the cytoplasmatic compartment - by monocytes through endocytosis involving clathrin-coated pits. It has previously been reported that the nucleotide-binding oligomerization domain (NOD)-like receptor (NLR) (nucleotide-binding domain leucine-rich repeat containing) family function as intracellular pattern-recognition receptors that sense various molecules [37]. Indeed this intracellular pathway which involves clathrin–dependent endocytosis leading to activation of mitogen activated protein kinases and NFkB activation [38] could be exploited by N10K for the direct induction of cytokine production. Because the presence of IL-6 is required for Th17 generation [39], the possibility that this Fc-peptide might favor Th17 activation arises.

Given that several chronic diseases such as rheumatoid arthritis are characterized by massive presence of autoantibodies, one could hypothesize that the synthetic peptides, potentially deriving from enzymatic cleavage of autoantibodies, via induction of IL-6, could help to trigger or maintain activation of Th17, which is considered a key cell population mediating many chronic inflammatory diseases [40].

It is worth noting that N10K is able by itself to induce NF-kB activation, suggesting that it induces a state of activation related to induction of IL-6 and an early induction of TNF-α production. Moreover, a late induction of IL-1β could be related to the increased early production of IL-6 and TNF-α. The specificity of this activity could be due to important differences in the signal pathway required for the induction of these cytokines [41], [42]. The regulatory activity of N10K is also manifested by a significant increase of selected PRRs. Indeed, a significant enhancement of Dectin-1 expression on N10K-treated monocytes was observed, however, no modulation of other PRRs such as TLRs was detected. Once again N10K selectively regulates some critical molecules of the immune response. Moreover, we observed that when N10K stimulation was associated with zymosan, which specifically binds to Dectin-1, a further increase in Dectin-1 expression was manifested.

Signalling from Dectin-1 following ligand binding is mediated through the cytoplasmic ITAM-like motif that becomes phosphorylated by Src family kinases, providing a docking site for Syk [43]. Signalling downstream from Syk involves CARD9, which assembles with BCL10 and MALT1 and has been identified as an essential downstream adaptor linking Syk-coupled receptors to NF-kB activation and, hence, cytokine production [21], [43], [44]. We showed that N10K uptake by monocytes results in enhancement of Syk, CARD9 and NF-kB activation induced by zymosan. These molecular events are correlated with a more intense production of proinflammatory cytokines when monocytes are pre-treated with N10K and then stimulated with heat inactivated *C. albicans*, curdlan or zymosan. Thus the importance of up-regulation of Dectin-1 is demonstrated by the important biological effects produced by N10K interaction with phagocytic cells. These results strengthen the hypothesis that N10K can improve the immune response to β-glucan Ags, therefore the anticandidal effect previously described [11] might be related to a direct antimicrobial effect associated with anti β-glucan immunostimulatory activity. This is also confirmed by the increased binding and internalization of heat inactivated *C. albicans* by N10K treated cells. Basically we can assume that N10K-induced immunostimulatory activity is closely related to the presence of β-glucan molecules, as a consequence fungal cells which contain β-glucan are the major end users of these effects.

Overall, our findings show that Fc-peptides potentially originating from Abs may, like CDR-related peptides, exert immunomodulatory activity, suggesting a new role for Abs in the immune response.
